# Potential for Modulation of the Fas Apoptotic Pathway by Epidermal Growth Factor in Sarcomas

**DOI:** 10.1155/2011/847409

**Published:** 2011-11-01

**Authors:** David E. Joyner, Kevin B. Jones, Stephen L. Lessnick, Joshua D. Schiffman, R. Lor Randall

**Affiliations:** ^1^Department of Orthopaedics, Huntsman Cancer Institute, The University of Utah, Salt Lake City, UT 84112, USA; ^2^Sarcoma Services, Center for Children, Huntsman Cancer Institute, The University of Utah, Salt Lake City, UT 84112, USA; ^3^Divison of Pediatric Oncology, The University of Utah, Salt Lake City, UT 84112, USA

## Abstract

One important mechanism by which cancer cells parasitize their host is by escaping apoptosis. Thus, selectively facilitating apoptosis is a therapeutic mechanism by which oncotherapy may prove highly advantageous. One major apoptotic pathway is mediated by Fas ligand (FasL). The death-inducing signaling Ccmplex (DISC) and subsequent death-domain aggregations are created when FasL is bound by its receptor thereby enabling programmed cell death. Conceptually, if a better understanding of the Fas pathway can be garnered, an oncoselective prodeath therapeutic approach can be tailored. Herein, we propose that EGF and CTGF play essential roles in the regulation of the Fas apoptotic pathway in sarcomas. Tumor and *in vitro* data suggest viable cells counter the prodeath signal induced by FasL by activating EGF, which in turn induces prosurvival CTGF. The prosurvival attributes of CTGF ultimately predominate over the death-inducing FasL. Cells destined for elimination inhibit this prosurvival response via a presently undefined pathway. This scenario represents a novel role for EGF and CTGF as regulators of the Fas pathway in sarcomas.

## 1. Introduction

Sarcomas, as cancers of mesenchyme, are rare and often quite deadly. Neoplastic processes arising in tissues of mesenchymal origin occur less frequently than those of ectodermal or endodermal origin, but behave in a very aggressive manner. In the United States, sarcomas have an annual incidence of over 10,000 cases per annum. Although relatively uncommon, these tumors as a group can behave in a nefarious fashion with currently reported mortality rates for certain subtypes greater than 50% [1]. As sarcomas arise in all parts of the body, especially the musculoskeletal system, the associated morbidity is substantially higher. Sarcomas inflict a tremendous emotional, physical, and financial toll on individuals and society alike. Furthermore, sarcomas affect patients of all ages, with 15% arising in patients younger than 15 years and 40% in patients older than 55 years. Accordingly, as the population ages, the incidence of these cancers will likely increase [2].

Aphoristically, the norm for a patient afflicted with metastatic sarcoma is death after an almost invective conflagration of mutilating surgery, radiation, and cytotoxic chemotherapy. Survival rates for stage IV sarcoma are 25% at best with this current triumvirate of clinical care [3]. Biotargeting has fueled limited optimism in the form of inhibitors of tyrosine kinases, mammalian target of rapamycin (mTOR), insulin growth factor receptor (IGFR), and histone deacetylase (HDAC) as well as others that selectively block prosurvival pathways [4]. Unfortunately, even with a more sophisticated inhibitory approach, many cancers circumvent a given blocked pathway to maintain a survival advantage and thus disadvantaging the patient. Facilitating apoptosis selectively in the cancer cell is another mechanism by which oncotherapy may prove highly advantageous. Unfortunately, detailed pursuit in understanding programmed cell death and the factors that influence it, even in the normal cell, has been a challenge. 

Fas/AP0-1/CD95 (tumor necrosis factor receptor superfamily, member 6) is the best characterized of the 29 receptors currently placed within the tumor necrosis factor (TNF) receptor superfamily. Fas induces apoptotic cell death when cross-linked with its membrane-bound ligand (FasL) [5], but can also demonstrate prosurvival properties by way of its interaction with signaling pathways that influence cell activation, proliferation, survival, and differentiation [6, 7]. Fas is expressed on a wide variety of cell types, including T cells and activated B cells, and plays a pivotal role in the regulation of the immune system [8]. Overexpression of Fas contributes to the pathogenesis of various malignancies, including lung cancer [9], colorectal cancer [10], breast cancer [11], and sarcomas [12, 13]. FasL is transcriptionally inactive in many cell types [14], but expression can be induced *in vivo *by DNA damage, or *in vitro *by serum deprivation [15]. Fas interaction with its ligand precipitates formation of the death-inducing signaling complex (DISC), which includes the adaptor protein Fas-associated death domain protein (FADD/MORT1), plus caspases 8 and 10 (CASP8/10) [14]. In type I cells, processed CASP8 directly activates downstream effector caspases (e.g., CASP3) leading to cell death, while in type II cells, activation of effector caspases depends on the CASP8-mediated cleavage of proapoptotic BID (BH3 interacting domain death agonist) and the subsequent release of mitochondrial proapoptotic proteins [16]. Recent studies have identified a second Fas-associated death domain protein: the death-domain-associated protein (DAXX). This 120 kD multifunctional protein modulates Fas-induced cell death via the signal-regulating kinase 1 (ASK1)-MEK-c-Jun-N-terminal kinase (JNK)/p38-Bax pathway [17]. DAXX also functions as a dominant negative inhibitor of transforming-growth-factor-beta- (TGF*β*-) induced apoptosis [18]. 

 In addition to regulating target cell lysis in the immune system, Fas signaling also contributes to the establishment of immune privilege and to tumor survival; therefore, the clinical and pharmacological potential of Fas and/or FasL as targets for therapeutic intervention has received considerable attention [19]. Although there are relatively nontoxic modulators of the Fas death pathway that are of potential clinical significance (i.e., interferon and nonsteroidal anti-inflammatory drugs—INSAIDs), systemic administration of rhFasL in humans was not considered feasible due to the Fas-mediated apoptosis of hepatocytes. Recent advancements in the development of FasL therapy, however, may enhance FasL efficacy. Compared to its membrane-bound counterpart, soluble FasL (sFasL) has a reduced capacity to activate Fas, even though all of the sequence information required for receptor activation is latently retained in the soluble ligand [20]. However, when bound to an appropriate antibody and administered as an antibody/ligand fusion protein, sFasL remains relatively inactive while enroute to the target, but once bound to the cancer cell, sFasL is converted into a fully active membrane ligand-like molecule [21]. Furthermore, combining the application of LCL 204, an inhibitor of the ceramide-metabolizing enzyme acid ceramidase, with FasL gene therapy sensitized head and neck squamous cell cancer cell lines to Fas-induced apoptosis both *in vitro* and in a xenograft model *in vivo *[22]. These new findings potentially open the door for renewed interest in employing FasL/Fas therapy for cancers.

Some TNF ligands reportedly bind to more than one member of the TNF superfamily of receptors [23], and crosstalk occurs at the level of intracellular signal transducers [24]. For example, FasL-induced cell death in tumor cells of epithelial origin is inhibited following activation of the epidermal growth factor receptor—EGFR/ErbB1 [25]. EGFR activation of the Akt pathway is required and sufficient for the anti-apoptotic function. EGF is a 6 kDa membrane protein that binds and activates its 170 kDa cell surface tyrosine kinase receptor. Extracellular ligand binding to the EGFR triggers a series of intracellular signaling cascades which collectively influence cell proliferation and survival, differentiation, and cell migration [26, 27]. Deregulation of signaling as a result of the aberrant expression of the EGFR has been implicated in oncogenesis [28]. The primary signaling pathways activated by EGF through the EGFR consist of (1) the extracellular signal-regulated kinase (ERK) mitogen-activated protein kinase pathway (MEK-ERK MAPK cascade), (2) the phosphatidylinositol-3 kinase PI3 K/Akt pathway, and (3) the signal transducer and activator of transcription (STAT) pathway [29–32]. Although present in normal cells, EGFR overexpression in tumor cells has been associated with a poor prognosis and decreased survival and EGFR activation is also involved in resistance to chemotherapy and radiation treatment [33]. EGF also reportedly protects rat prostatic epithelial cells from TGF*β*1-induced cell death [34]. TGF*β*1 (transforming growth factor beta 1), like EGF, can modulate the FasL/Fas apoptotic pathway. TGF*β*1 inhibits Fas-induced cell death in pre-B-cells by blocking the PI3K pathway [35]. TGF*β*1 can also induce apoptosis through the FasL-independent activation of the Fas death pathway in human gastric SNU-620 carcinoma cells [36]. 

In view of the above, we suggest a relationship exists between growth factor activity and modulation of the Fas apoptotic pathway in mesenchymal tumors. In 1999, Gibson et al. [25] reported that EGF protected epithelial cells from Fas-induced apoptosis. Studies published by this laboratory [37, 38], as well as our ongoing research, suggest a similar inhibitory effect by EGF, but in mesenchymal cells. Sarcoma transcriptional patterns imply the following scenario. Viable cells within an untreated primary sarcoma are exposed to FasL. To counter the prodeath signal induced by FasL, and thus, to maintain viability, the cells respond by inducing EGF, which in turn activates (directly or indirectly) prosurvival connective tissue growth factor (CTGF). The prosurvival attributes of CTGF ultimately predominate over the death-inducing FasL. Cells destined for apoptotic elimination inhibit this prosurvival response. We now propose three genes—FasL, TGF*β*1, and CTGF—are intimately involved in the regulation of the Fas death pathway, and their coordination and respective contributions are modulated by EGF in tumors of mesenchymal origin. Our tumor and *in vitro* data strongly support a role for growth factor modulation of Fas-induced cell death, possibly through EGFR activation of the MAPK signal transduction pathway. This scenario represents a novel role for EGF as a regulator of Fas-induced cell death in mesenchymal neoplasms. 

## 2. Materials and Methods

### 2.1. Sarcomas

Eighty-two primary sarcomas were acquired from three institutions and processed according to IRB-approved and HIPAA-compliant protocols. The 82 sarcomas represented the 57 tumors used in our original evaluation of the Fas death pathway [38], plus an additional 25 sarcomas included specifically for this analysis. Total RNA was extracted and purified from the homogenized tissues using RNeasy kits supplied by Qiagen (Germantown, Md, USA), or by TRIzol according to the manufacturer's recommendations (Invitrogen, Carlsbad, Calif, USA). RNA quality was evaluated using an Agilent Bioanalyzer (Santa Clara, Calif, USA). Sarcoma histotypes (number of tumors/histotype) consisted of chondrosarcoma (5), Ewing's sarcoma (EFT-8), fibrosarcoma (1), hemangiopericytoma (2), leiomyosarcoma (7), liposarcoma (3), malignant fibrous histiocytoma (MFH-5), malignant peripheral nerve sheath tumor (MPNST-9), myxofibrosarcoma (3), osteosarcoma (7), rhabdomyosarcoma (4), synovial sarcoma (9), and “unclassified” sarcoma (19).

### 2.2. Cell Culture

SW1353 chondrosarcoma cell cultures (ATCC: HTB-94) in Leibovitz's L-15 medium (Invitrogen) containing 10% fetal bovine serum (FBS; Hyclone, Logan, Utah, USA) were maintained in mid-log phase within a 37°C humidified incubator containing 5% CO_2_. Culture medium containing FBS is referred to hereafter as “complete medium.” For experiments, a predetermined number of log-phase SW1353 cells were inoculated in complete medium into 25 cm^2^ tissue culture flasks one day prior to treatment. Cell cultures were subsequently washed, serum-starved (serum-free; SF) for 24 h, and then stimulated with 5 or 25 ng/mL recombinant human EGF (rhEGF; E9644; Sigma) in SF medium at 37°C for an additional 1 h or 24 h. Control flasks received SF L-15 medium only. Following treatment, cultures were washed and SF medium was added to each flask. Cultures were incubated at 37°C for an additional 24 h prior to processing for real-time RT-PCR. Total RNA was extracted from cells using RNeasy kits (Qiagen). Complementary *in vitro *experiments were analyzed for EGF-induced gene expression when the cultures were maintained and treated in complete L-15 medium. A minimum of three separate experiments with three flasks per treatment dose were conducted for both SF and complete medium protocols. To determine the effect of receptor inhibitors on gene expression, SW1353 cultures were pretreated for 24 h with the EGF receptor (EGFR) inhibitor AG1478 (tyrphostin; T4182; Sigma), alone at 200 nM, or in combination with 200 nM TGF*β* receptor inhibitor SB431542 (Tocris Biosciences, Ellisville, Mo, USA). Following the initial 24 h inhibitor treatment, cultures were stimulated for an additional 24 h with rhEGF coupled with the receptor inhibitor(s) prior to being assayed for gene expression. 

### 2.3. Real-Time RT-PCR Assays for Gene Expression

Tumor and SW1353 cell mRNA content was quantified by real-time RT-PCR using TaqMan Gene Expression Assays and the ABI PRISM 7900 HT Sequence Detection System with related software (Applied Biosystems, Foster City, Calif, USA). Glyceraldehyde-3-phosphate dehydrogenase (GAPDH) served as the reference standard. Individual samples were screened for the Fas-related genes EGF, TGF*β*1, FasL, Fas, CTGF, FADD, DAXX, SMAD3 (Mothers against decapentaplegic homolog 3), SMAD7, CASP8, CASP10, and CFLAR (CASP8 and FADD-like apoptosis regulator). Real time RT-PCR cycle scores were normalized by subtracting the sample GAPDH cycle number from each gene cycle number. 

### 2.4. Enzyme-Linked Immunosorbent Assay (ELISA) and Dot Blotting

We quantified by ELISA protein concentrations (pg/mL) of EGF, FasL (R&D Systems, Minneapolis, Minn, USA), and TGF*β*1 (YES Biotech Laboratories, Ltd, Mississauga, ON, Canada) in 21 of the 82 sarcomas used in this study. The 21 tumors represented the only sarcomas in the 82-tumor cohort whose soluble proteins were available for use. We used a standard dot blot assay to document the presence or lack of CTGF protein in the 21 sarcoma samples since commercial ELISA kits for CTGF are currently unavailable. Tumor-soluble proteins were isolated from the 21 sarcoma samples using TRIzol and then quantified by spectrophotometry using the BCA protein assay kit provided by Thermo Scientific (Rockford, Il, USA). A colorimetric, cell-based ELISA kit (RayBio Cell-Based EGFR (activated) ELISA kit; RayBiotech, Inc., Ga, USA) was used to verify EGFR activation following rhEGF stimulation of the SW1353 chondrosarcoma cell line. 

### 2.5. Statistical Analysis

Statistical analyses were performed using the Microsoft Office Excel 2003 statistical analysis tool option as described previously [37, 38]. Briefly, the GAPDH-normalized RT-PCR scores were tested for randomness and normality (http://home.ubalt.edu/ntsbarsh/stat-data/Javastat.htm) prior to the parametric analysis. The ABI PRISM 7900 HT sequence detection system reports an mRNA concentration as “the cycle when amplification of the target is first detected; the higher the starting copy number of the nucleic acid target is, the sooner a significant increase in fluorescence (i.e., the smaller the cycle number) is observed.” Cycle values for our tumor and cell culture samples ranged from 16 to 39. In order to accommodate the analysis, we arbitrarily assigned a cycle value of 40 to individual samples lacking detectable levels of mRNA. Forty represented one cycle greater than the highest cycle value produced by the lowest detectable level of mRNA (i.e., cycle 39). In view of the fact that we lacked a reference plasmid of known size, with known copy number, which would have been used to define constituent copy numbers of transcripts, our results are reported herein as cycle numbers, rather than as absolute copy number. Ten Fas-related genes were assayed for mRNA content in SW1353 cell cultures. FasL was not detected in any of the cell lysates, and DAXX and FADD were not assayed. Multiple regression using the least squares method was used to identify significant predictor variables, based on selected response variables. Coexpressed genes were identified using Pearson's correlation coefficient. Gene pairs whose mRNAs correlated across a cohort of samples were considered to be coexpressed [39]. Correlation probability values were adjusted using the Bonferroni adjustment for multiple tests. Univariate analysis of variance (ANOVA) and *t*-tests compared transcript concentrations within and among histotypes and for cell culture treatments. Scatter plots were generated to ensure the data sets were not unduly influenced by outliers (data not shown). 

## 3. Results and Discussion

### 3.1. Transcript Prevalence in Untreated Primary Sarcomas

We quantified transcript levels in untreated primary sarcomas using real-time RT-PCR in order to identify coexpression patterns within the Fas death pathway. The mean RT-PCR cycle number for each of 11 genes when averaged over all 82 sarcomas ranged from 3.1 for CTGF to 12.6 for EGF, equivalent to approximately a 2^10^ difference in mRNA content. Theoretically, the amount of DNA (derived from mRNA) doubles with every cycle of PCR during the exponential phase; therefore, an 8-fold difference in mRNA template would be approximately 3 cycles apart. Of the twelve genes evaluated, only EGF (*P* = 2.6*E* − 08; mean cycle number for 11 histotypes = 12.6; range = 8.0–16.4), FasL (6.7*E* − 07; mean = 12.3; range = 9.8–15.1), and CTGF (2.8*E* − 03; mean = 3.1; range = 1.6–5.6) demonstrated significant differences (*P* ≤ 0.01) in mRNA content among the 11 sarcoma histotypes evaluated; fibrosarcoma with a sample ize of one was not included within the ANOVA. 

In contrast to other tumor types, (e.g., carcinomas [40]), sarcomas frequently exhibit little or no EGF mRNA and/or protein, when screened by PCR or immunohistochemistry [41]. Our 82-sarcoma cohort was no exception. EGF transcripts were detected at low concentrations in 75 of the 82 sarcomas. The highest EGF concentrations (i.e., lowest cycle numbers) were recorded for hemangiopericytomas (mean cycle number = 8.0, *n* = 2) and Ewing's sarcomas (mean = 8.8, *n* = 8), while the lowest levels were in synovial sarcomas (mean = 16.4, *n* = 9). Of the seven sarcomas lacking detectable levels of EGF, only two were synovials. EGF cycle numbers for individual tumors ranged from 5.9 to 17.3, equivalent to a 2^12^ difference in mRNA content among the 82 tumors. 

High FasL cycle numbers also reflected very low FasL mRNA concentrations in all 82 sarcomas. FasL and EGF concentrations were significantly (*P* ≤ 0.001, ANOVA) lower than the transcript levels of the other genes inventoried in this study. Maximum FasL was detected in malignant fibrous histiocytomas (mean cycle number = 9.8, *n* = 5) and in MPNST (mean = 10.6, *n* = 9), while the lowest quantities were in synovial sarcomas (mean = 15.1). Inducible or constitutively expressed FasL has been reported for a variety of tumor types, including sarcomas [42, 43]. FasL protein presumably functions to enhance tumor survival via immune privilege, although this function has been challenged, for example [44]. FasL mRNA and protein have also been detected in tumor cell lines, although Ryan et al. [45] cautioned against using FasL mRNA alone as an indicator of FasL expression. Apparently, cells in culture can maintain constitutive expression of FasL protein in spite of extensive variations, or even the lack of FasL mRNA production. In our study, FasL transcripts were not detected in SW1353 cells when cultured in complete medium. However, FasL became transcriptionally and translationally active in SW1353 cultures, as it does in other cell types [15], when the cultures were deprived of serum for extended periods of time.

 CTGF was present in all tumors examined and was particularly abundant in MPNST (mean cycle number = 1.6, *n* = 9), leiomyosarcomas (mean = 1.6, *n* = 7), and chondrosarcomas (mean = 1.7, *n* = 5). We expected CTGF concentrations to be high in chondrosarcomas [46], but in fact, only one of the seven tumors with the highest levels of mRNA was a chondrosarcoma, while the others consisted of (two tumors each) MPNST, leiomyosarcoma, and osteosarcoma. 

The remaining nine genes, all of which generated mean cycle numbers ranging from 3.9 (TGF*β*1) to 7.4 (CASP8 and CASP10), differed little in mRNA content among histotypes (*P* ≥ 0.01). These nine genes also showed very low variance estimates for mRNA content, ranging from 1.6 for CASP8 to 3.9 for Fas. In comparison, variance values for EGF, FasL, and, CTGF were 10.0, 7.6, and 6.6, respectively. 

Our results verify transcription of all 12 Fas-related genes in sarcomas and suggest a majority of these genes vary little in expression level (constitutive expression?) within or among sarcoma histotypes. Expression of the facultative genes FasL and EGF, on the other hand, varied significantly within and among histotypes. EGF was the only gene whose mRNA was not detected in all 82 tumors. 

### 3.2. Coexpression in Tumors and SW1353 Cells

Gene coexpression analysis is based on the premise that genes with similar functions have similar expression patterns [47]. Coexpression data sets, typically derived from microarray studies, are used to generate coexpression networks which reflect the interdependence of molecules, cellular processes, and regulatory pathways [48]. This analytical approach has been used to delineate functional gene sets in rice (*Oryza*) [49], stress response [50], and cancer [51] and to identify orthologous genes among distantly related taxa [52]. 

Coexpressed tumor genes, defined in this study using Pearson's correlation coefficients [39], are identified as bold correlation coefficients in Table 1. Coexpression analysis of the 82-tumor cohort indicated strong associations between Fas and six of the seven recognized participants in the Fas death pathway (DAXX, SMAD3, SMAD7, CASP8, CASP10, and CFLAR) [53, 54], as well as associations among those six genes. Interestingly, neither FasL nor EGF correlated with Fas, while CTGF and TGF*β*1 did. We reported strong correlations between Fas, TGF*β*1, and/or CTGF previously [37, 38]. Also noteworthy is the fact that DAXX, rather than FADD correlated with Fas, as well as with SMAD3, both caspases, and CFLAR. FADD is considered the primary adaptor molecule in the Fas death pathway [55], while the implementation of Fas-induced apoptosis via DAXX remains controversial [56, 57]. Ironically, although DAXX acts as an intermediary to convey proapoptosis signals from the TGF*β* receptors in AML12 hepatocyte and CH33 immature B-cell lymphoma [18], DAXX did not correlate with TGF*β*1 in our sarcoma samples. Perhaps a correlation between DAXX and TGF*β* would have been evident had we included one or both TGF*β* receptors rather than TGF*β*1.

To determine whether coexpression patterns apparent in the 82-tumor cohort could be induced *in vitro* with rhEGF, we used the SW1353 chondrosarcoma cell line to support our contention that EGF modulates the Fas pathway. A colorimetric, cell-based ELISA kit was used to verify EGFR activation following rhEGF stimulation. SW1353 cells tested positive for EGFR and cells stimulated with rhEGF and assayed with an *α*-phospho-EGFR (activated) antibody displayed significantly higher (*P* ≤ 0.05) optical density readings than untreated (control) cells, thus confirming activation of the EGFR following rhEGF stimulation.

The microenvironment is certainly an influential factor on all cellular activity. To assess environmental stress on the cell, we compared the coexpression profiles of untreated control cultures in complete medium (*n* = 23) and serum-free (SF) conditions (*n* = 18), plus rhEGF-induced cultures (*n* = 34) against the gene associations apparent in tumors. The complete medium control cultures consisted of cells grown under “maintenance” conditions (i.e., exponential proliferation). SF control cultures were included for two reasons: (1) tumors *in situ* reportedly contain quiescent as well as rapidly proliferating cell populations and serum deprivation (SF) *in vitro* induces cellular quiescence [58]; (2) growth factors are typically applied *in vitro* under SF conditions following a minimum 24 h serum deprivation treatment [59], and extended serum deprivation (≥17 h) has a significant impact on gene expression [60]. Treated cultures included both rhEGF and receptor-inhibited/rhEGF-induced samples and were incorporated into a single “treated” cohort in order to include within the analysis a wide variety of potential physiological states, any combination of which might influence coexpression. The correlation matrices representing nine genes assayed in the two control cohorts are provided in Table 2, while Table 3 lists correlation coefficients for those same nine genes in treated cultures.

The most prominent difference among the *in vitro* and tumor correlation matrices involved EGF. EGF lacked correlation in the 82-tumor cohort and in SF control cultures, but correlated with Fas-related genes in rhEGF-induced cultures and in the complete medium controls, although the coexpression patterns differed remarkably between these two responsive cohorts. In particular, EGF correlated with TGF*β*1, CTGF, Fas, and SMAD3 in the complete medium cohort (Table 2), but not in the rhEGF-induced cohort (Table 3), while EGF associated with SMAD7, both caspases and CFLAR in rhEGF-induced cells, but not in the complete medium controls. This circumstance might have been in response to the composition of the serum supplement, which presumably included growth factors, or may have been induced by the *in vitro* treatment and harvest schedule employed in this study. We cannot explain at this time why EGF lacked correlation in the tumor cohort and in SF controls (although see below).

Three coexpression patterns remained consistent in all four cohort matrices: (1) CASP8 and CASP10 correlated strongly with each other, and both with CFLAR; (2) Fas correlated with SMAD3; (3) CTGF and TGF*β*1 correlated with Fas. The associations of these respective genes may be so tightly intertwined and controlled that the genes remained refractory to the *in vitro *modulations utilized in this study. Surprisingly, TGF*β*1 lacked correlation with CTGF and SMAD3 in the rhEGF-induced matrix, in stark contrast to their strong correlations in the other three matrices. It is well established that CTGF is induced by TGF*β* in mesenchymal cells [61] and that SMAD3 is directly responsive to TGF*β* stimulation in a variety of cell types [62], so correlations among TGF*β*1, CTGF, and SMAD3 were expected in all four matrices. A direct association between EGF and CTGF was demonstrated by Samarakoon et al. [63] when they inhibited the EGFR with AG1478, a receptor tyrosine kinase inhibitor which preferentially blocks EGFR kinase without reducing expression of EGFR [64], which blocked CTGF expression in vascular smooth muscle cells. It is possible that collation of samples pretreated with TGF*β* and EGF receptor inhibitors within the rhEGF cohort is responsible for the lack of association between TGF*β*1, CTGF, and SMAD3 in the rhEGF-treated samples.

Lastly, a number of gene associations highlighted in the tumor matrix were not replicated *in vitro*. This was particularly true for Fas itself. The lack of correlation between Fas and genes downstream of Fas in all three *in vitro* matrices could also be an artifact of the treatment and harvest schedule used for *in vitro* experiments. 

In additional to the 24 h rhEGF *in vitro* experiments, we also screened for coexpression in cell cultures stimulated for 1 h with 5 ng/mL rhEGF, without receptor inhibition, to further demonstrate the modulatory influence of EGF on gene transcription. In these experiments, relevant gene pairs correlated significantly in rhEGF-stimulated cultures, but not in untreated control cultures (Table 4). FasL mRNA was not expressed in treated and control cultures; therefore, it was not included in this analysis. 

EGF induces cell growth, migration, invasiveness, and survival in tumors and in cultured cells [65, 66]; however, the mechanisms involved remain unclear. Our coexpression analysis complements suggestions made by others that genes within the Fas pathway respond to EGF stimulation [67–69]. Our coexpression analysis does not, however, clarify whether the response is prosurvival or prodeath, nor does it identify which potential Fas ligands were functional. On the other hand, the analysis laid the foundation for development of an EGF/CTGF/Fas model, described below, that suggests a prosurvival function for EGF in mesenchymal cells. 

### 3.3. Fas Ligand Functionality in Sarcomas and SW1353 Cultures

We recently published evidence supporting a modulatory influence by EGF on transcription within the Fas apoptotic pathway in sarcomas [38]. Ten Fas-related genes were assayed by real-time RT-PCR in a cohort of 57 primary sarcomas. The 57 sarcomas were arbitrarily sorted into two subcohorts based on tumor EGF mRNA content. Multiple regression analysis employing FasL and TGF*β*1 as predictor variables, and Fas as the response variable, indicated that TGF*β*1 expression predicted Fas mRNA concentrations in the low-EGF subcohort, while FasL predicted Fas content in the high-EGF subcohort. We concluded that Fas ligand functionality correlated with, and may be modulated in, sarcomas by EGF. 

In our current analysis involving 82 primary sarcomas, 57 of which were used in the original analysis [38], we included EGF and CTGF as predictor variables within the Fas/FasL/TGF*β*1 multiple regression model. Inclusion of CTGF was based on evidence published previously by this lab that showed a very strong correlation between CTGF and Fas mRNA content in sarcomas with low-EGF content, which lacked correlation in sarcomas with higher-EGF content [37]. When the relationships between Fas and the four predictor variables were analyzed over the 82-tumor cohort, CTGF and FasL predicted Fas mRNA content, while TGF*β*1 and EGF lacked predictive value (Table 5). Removal of EGF and CTGF from the regression model showed FasL and TGF*β*1 predicted Fas for the 82-tumor cohort equally well (data not shown). Division of the 82 tumors into two EGF-related subcohorts of 41 tumors each, and with EGF and CTGF removed from the regression, produced results consistent with our previous findings [38]. These results imply the association between Fas and TGF*β*1 may be coordinated through CTGF. Surprisingly, EGF, which lacked predictive value for the 82-tumor cohort and was not included as a predictor variable in [38], became a significant predictor of Fas when the 82 tumors were arbitrarily divided into the two EGF-related subcohorts and CTGF was included within the analysis (Table 5). 

CASP8 and CASP10 function downstream of Fas, and activation of *CASP8* (and probably *CASP10*) via the Fas death-inducing signaling complex is a requirement for Fas-induced cell death. We questioned whether the associations involving Fas and its potential ligands described above would carry over to downstream genes. Of the four predictor genes evaluated, TGF*β*1 predicted CASP8 and CASP10 content in the 82-tumor cohort, but only marginally, while FasL effectively predicted CASP10 but was marginal for CASP8 (Table 5). Neither CTGF nor EGF successfully predicted caspase content. With division of the tumor cohort into the two EGF-related subcohorts, we expected FasL to dominate in the high-EGF subcohort and CTGF to provide predictive power in the low-EGF subcohort. Our regression model suggested FasL and/or TGF*β*1 could both be functional in the high-EGF subcohort (*P* ≤ 0.01), but FasL, rather than CTFG or TGF*β*1, was significant for the low-EGF cohort, and then only for CASP10 (*P* = 5.9*E* − 03). Our interpretation of these results is that EGF and CTGF are “prosurvival” genes and, therefore, may correlate with, but do not necessarily induce, these two caspases. FasL and TGF*β*1, on the other hand, activate prodeath CASP8 and CASP10 via Fas.

To evaluate these same gene associations in cultured cells, SW1353 cultures were stimulated with rhEGF for 24 h and harvested for RT-PCR. The predictive values of EGF, TGF*β*1, and CTGF for Fas and both caspases are also provided in Table 5. FasL was not included in this analysis. TGF*β*1 was the only significant predictor of Fas in SF controls and rhEGF-treated cultures, while CTGF filled that role for cultures maintained in complete medium. Both TGF*β*1 and CTGF retained predictive value for caspases in complete medium control cultures, but neither TGF*β*1 nor CTGF showed predictive value for caspases in SF controls or in rhEGF-treated cultures. Collectively, trends in the *in vitro* data support our tumor analysis. However, the lack of FasL expression by control and treated cell cultures undoubtedly affected the molecular coordination of the three other genes included as predictor variables within the regression model. This in turn made it difficult to clearly discern patterns that were statistically attributed to EGF activity in the sarcomas. In addition, the range of EGF mRNA concentrations within the *in vitro* cohorts varied little between and among samples. The lack of variation in EGF expression made it impossible to explore the impact of EGF content on expression patterns.

In view of the tumor and *in vitro* results described above, we propose that three genes—FasL, TGF*β*1, and CTGF—are intimately involved in the regulation of the Fas death pathway, and their coordination and respective contributions are modulated by EGF in sarcomas. Therefore, based on our collective findings, we suggest a novel role for EGF as a co-regulator of the Fas pathway in mesenchymal neoplasms. 

### 3.4. Derivation of the EGF/CTGF/Fas mRNA Model

**Figure 1:**
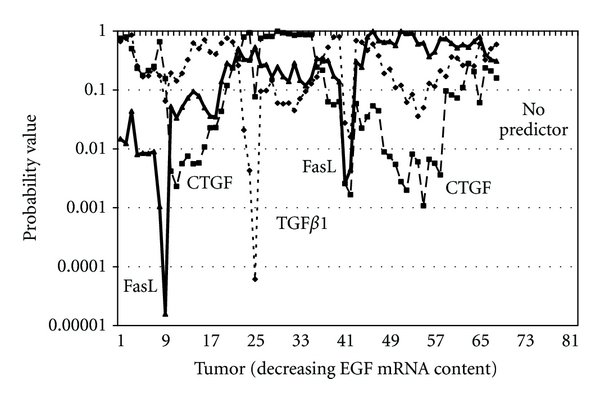
Probability indices for predictor variables FasL, TGF*β*1, and CTGF regressed against Fas in 82 primary sarcomas.

Growth factors are mitogens [70]. Apoptosis, on the other hand, is programmed cell death. Given the opposing functional roles of proliferation and apoptosis, these two processes must be coordinated. We propose that EGF contributes to that coordination in sarcomas by regulating the expression and/or functionality of the proapoptotic and/or anti-apoptotic proteins: FasL, TGF*β*1, and CTGF. The possibility of “crosstalk” among *EGF*/*TGF*β*1*/*CTGF*/*FasL* and interactions between EGF/TGF*β* receptor signaling and the Fas death pathway have been reported previously [34]. To develop our EGF/CTGF/Fas model, we ranked the 82-sarcoma cohort by descending EGF mRNA content. We then used a series of partially overlapping, 15-tumor, multiple regressions that collectively incorporated all 82 tumors in order to identify which predictor variable(s)—FasL, TGF*β*1, and CTGF—best described Fas content within each 15-tumor cohort. The first regression encompassed tumors number 1 through number 15, the second regression incorporated tumors number 2 through number 16, and so forth. The final regression analyzed 15 tumors containing little or no EGF mRNA. The resulting probability “indices” for the three potential Fas ligands are illustrated in Figure 1. For this analysis, we view the resulting probabilities as indices, rather than as probability values *per se*, due to the statistical requirements associated with the testing of overlapping samples [71]. 

Our mRNA model formulates testable predictions regarding Fas ligand functionality in relation to tumor EGF content. The model suggests FasL may be the primary, if not the only, functional Fas ligand in mesenchymal tumors supporting high concentrations of EGF mRNA, since it was the only ligand of the three with indices ≤0.05 for the sarcomas within this EGF range. As the EGF mRNA content of tumors decreases, FasL is replaced by CTGF as the “best predictor,” which is replaced in turn by TGF*β*1. There appears to be a second abbreviated tumor zone represented by tumors number 41 through number 56 where FasL again predicts Fas content. This zone is overlapped and extended substantially by a second CTGF zone (tumors number 44 through number 73). The Fas concentrations in tumors number 74 through number 82, which contained little or no EGF mRNA, could not be predicted by the three potential ligands. The transition from one potential ligand to the next is gradual and overlapping. 

**Figure 2:**
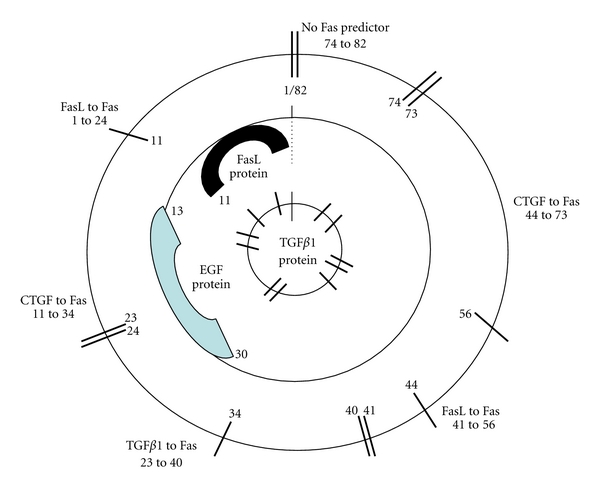
EGF/CTGF/Fas mRNA model.

The presumed interrelationships among EGF, Fas, and the three potential Fas ligands are depicted in our EGF/CTGF/Fas model (Figure 2). We have included within this schematic tumors that contained FasL, EGF, and TGF*β*1 proteins, as defined by ELISA. In the schematic, all three circles represent the same tumor continuum, starting with tumor number 1 which had the highest concentration of EGF mRNA and ending with tumor number 82, which lacked EGF mRNA. The hash marks through the outer circle delimit the presumed functional distribution of each potential Fas ligand (i.e., range of successive tumors for which the index for that variable was ≤0.05, as shown in Figure 1). For example, FasL predicted Fas mRNA content in tumors number 1 through number 24, CTGF in tumors number 11 through number 34, and so forth. The middle circle identifies the tumors containing FasL (tumors number 1 through number 11) or EGF (number 13 through number 30) protein. The hash marks through the small, inner circle represent the 11 tumors that contained TGF*β*1 protein. We derive three predictions from the mRNA model: (1) FasL protein induces EGF transcription; (2) EGF subsequently modulates TGF*β*1 and CTGF expression and activity; (3) CTGF defines TGF*β*1 activity, which ultimately favors cell survival over cell death.

EGF promotes cell survival, and it is our hypothesis that EGF signaling is one mechanism used by sarcomas to negate FasL-induced apoptosis. A particularly relevant finding regarding this topic was reported by Reinehr et al. [72]. They found for quiescent hepatic stellate cells (HSCs), which are typically resistant to FasL-induced apoptosis, that FasL, generally considered to be a prodeath molecule, stimulated EGFR signaling, which in turn enhanced HSC survival. In other words, in quiescent HSCs, FasL and other death receptor ligands function as mitogens. The process is EGF dependent and involves a protease-mediated liberation of EGF, which subsequently activates the EGFR. Activation of the EGFR in quiescent HSC involves phosphorylation of the EGFR tyrosine residues 845, 1173, and 1045. Phosphorylation of tyrosine 1045 mediates EGFR internalization. In hepatocytes, FasL also activates the EGFR, but the process is not ligand dependent, nor is the EGFR internalized. The proposed pathway in hepatocytes involves FasL activation of p47^phlox^, a regulatory subunit of NADPH oxidase. The resulting oxidative stress induces the Src family kinase Yes, which in turn induces EGFR phosphorylation at Tyr^845^ and Tyr^1173^. Tyr^1045^ is not phosphorylated, hence no EGFR internalization. The reactive oxygen species (ROS) signal activates JNK, which is a requirement for Fas-related apoptosis [73]. The JNK pathway is not activated in FasL-stimulated quiescent HSC. 

The mRNA samples used in our tumor analysis were derived from untreated sarcomas which presumably contained both quiescent and proliferating cell populations. Solid tumors frequently contain a significant proportion of quiescent cells. The sarcoma mRNA indices, which defined our model, may typify the quiescent populations within those tumors. Since the tumors were not exposed to radiation or chemotherapy prior to resection, each sample should have typified a tumor that was undergoing routine tumor maintenance (progression/stasis) at the time of resection. Accordingly, the presumptions guiding the formulation of our mRNA model include the following: (1) the mRNA probability indices generated by the multiple regression analysis reflect the functional distributions of the respective genes, alone or in combination with other genes; (2) the 82 sarcomas used to develop the model had all been exposed to FasL protein within a presently undefined time frame; (3) by ranking the tumors according to descending EGF mRNA content, we mapped incremental “steps” in the physiological response of tumors to FasL exposure; (4) the three potential Fas ligands are modulated by EGF; (5) for this particular apoptotic pathway, EGF defines whether a cell lives or dies by regulating TGF*β*1 and/or CTGF; (6) the EGF-induced response to FasL is universal among mesenchymal cells (i.e., transcends tumor cell-of-origin and benign versus malignant). 

We believe this approach allows us not only to evaluate the interrelationships between response and predictor variables within each 15-tumor cohort, but also to visualize progressive changes in the respective contributions of predictor variables attributable to incremental reductions in EGF mRNA. Fundamental to this approach is the assumption that transcription reflects the physiological state of the untreated tumors at the time of resection and is not contingent upon, nor specific to, tumor histotype or cell of origin. Transcript modulation by microRNAs may be partially responsible for this. We consider our ranking of tumors by descending EGF mRNA content in order to evaluate collectively the influence of EGF on gene transcription within the Fas death pathway as analogous to viewing successive frames of a 35 mm movie. Each tumor (frame) provides a time-specific “snapshot” of the physiological state of that tumor (content of the movie), but by itself may not provide sufficient information to discern a sarcoma-wide transcriptional response (theme of the movie). It is only when all of the tumors (frames) representing multiple histotypes are combined into a single cohort, ranked by decreasing EGF content, and then viewed in its entirety (i.e., in context) that the transcriptional response (theme) becomes apparent. 

### 3.5. Tumor EGF and FasL Protein Expression Conform to the mRNA Model

**Figure 3:**
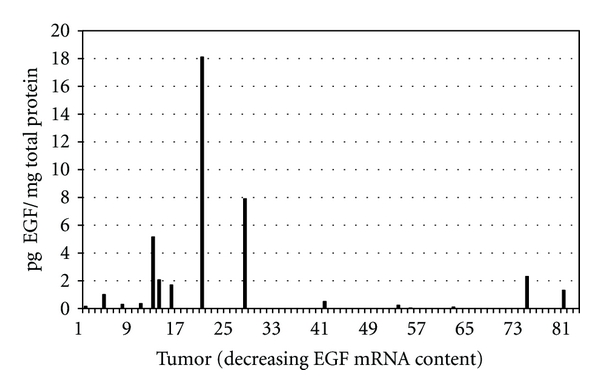
Distribution of EGF protein in 21 sarcomas included within the 82-sarcoma cohort.

Our model is based on tumor mRNA content. Arguably, the ligand functionality indices illustrated in Figure 1 could represent one of an infinite number of possible patterns generated through the randomization of the 82 sarcomas. However, we believe our model makes testable predictions regarding protein expression, and their confirmation will strengthen our hypothesis. We propose that sarcomas with the highest EGF mRNA concentrations represent tumors stimulated to undergo EGF transcription. Transcription is repressed subsequently and ultimately terminated when adequate EGF protein becomes available to carry out the prescribed function(s) or the EGF-mediated response is no longer necessary. If our mRNA model is correct, EGF protein should be present in sarcomas with high(er) levels of EGF mRNA, but not necessarily in tumors with the highest concentrations of EGF mRNA, and reduced in content or lacking in sarcomas with little or no EGF mRNA. To test this prediction, we quantified by ELISA the EGF protein content in 21 of the 82 sarcomas used for this proposal. The 21 tumors represented the only sarcomas in the 82-tumor cohort whose soluble proteins were available for use. The EGF mRNA concentrations of the 21 tumors spanned the entire range of EGF concentrations represented within the 82-tumor cohort. A majority of the sarcomas with detectable levels of EGF protein contained high concentrations of EGF mRNA (Figure 3). The primary EGF-protein-containing tumors are labeled number 13 through number 30 in Figure 3. Six of the 21 sarcomas lacked detectable levels of EGF protein; all six had EGF mRNA concentrations below the level of tumor number 30. The EGF protein pattern conforms to our model.

**Figure 4:**
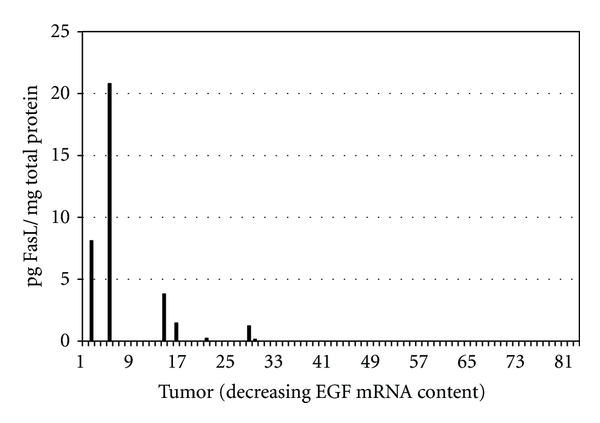
Distribution of FasL protein in 21 sarcomas within the 82-sarcoma cohort.

We then quantified FasL protein concentrations in the same 21 sarcoma samples. FasL mRNA was detected in all 82 sarcomas, at concentrations equivalent to EGF mRNA content. Our mRNA model predicts that FasL is functional in high-EGF tumors, but may also show limited activity in the mid-EGF range. Initially, we assumed FasL protein would predominate in tumors that also contained high concentrations of FasL mRNA, in a manner analogous to EGF. This proved not to be the case. When the 82 sarcomas were ranked by decreasing FasL mRNA concentration, rather than by decreasing EGF, the five FasL-protein-bearing tumors were distributed across the entire 82-tumor cohort (represented by tumors number 9, number 25, number 57, number 58, and number 79). On the other hand, when the 82 tumors were ranked by decreasing EGF mRNA content (Figure 4), FasL protein was detected only in tumors with high EGF mRNA concentrations. This protein pattern conforms to our mRNA model since the distribution of FasL-protein-bearing sarcomas along the 82-tumor EGF mRNA gradient overlaps the tumor zone where FasL is the only significant predictor of Fas mRNA content (refer to Figure 1). The protein analysis highlights four important points regarding FasL in mesenchymal tumors: (1) EGF and FasL mRNA concentrations did not correlate in the 82 primary sarcomas; therefore, why is FasL protein present only in sarcomas containing the highest concentrations of EGF mRNA? Might FasL protein induce EGF expression in mesenchymal tissues, as was the case for a variety of EGFR ligands induced by FasL in human epidermis [74]? (2) Why was there no discernable association between FasL mRNA concentration and the presence of FasL protein? A lack of correlation between FasL mRNA and protein expression was also reported by Ryan et al. [45]. (3) Is the stability of the FasL protein influenced by EGF protein (i.e., FasL-protein-bearing tumors were “upstream” of a majority of the tumors containing EGF protein)? (4) Does the lack of EGF protein render cells functionally receptive to FasL transcription and translation? Since stimulation of human epidermal carcinoma A431 cells with EGF induces FasL expression [75], we suggest EGF may also impact FasL in mesenchymal cells. 

### 3.6. TGF*β*1 Protein Expression Deviates from the mRNA Model, While CTGF Conforms to the Model

**Figure 5:**
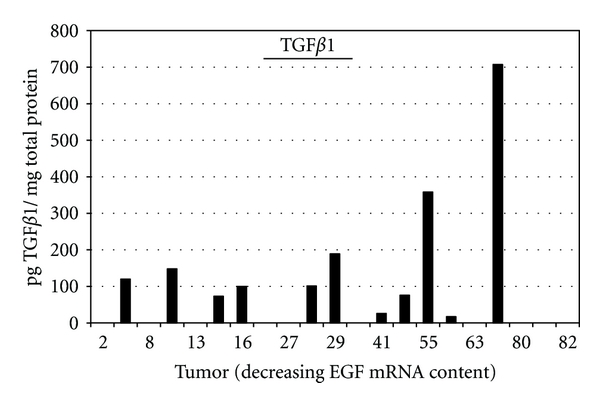
Distribution of TGF*β*1 protein in 21 sarcomas within the 82-sarcoma cohort. The line labeled TGFbeta1 overlays the tumors within which TGF*β*1 significantly predicted Fas mRNA content.

We propose that TGF*β*1 and CTGF are principal participants within the Fas death pathway, and their respective contributions and potential cross-interactions within the pathway are modulated by EGF. TGF*β*1 mRNA significantly predicts Fas mRNA content in tumors number 23 through number 40, while CTGF dominates in tumors number 11 through number 34, and again in tumors number 44 through number 73 (Figure 1). We used a commercially available ELISA kit to quantify TGF*β*1 protein concentrations in the 21 sarcoma samples described above, but used a dot blot assay to document the presence or lack of CTGF protein in the samples, since commercial ELISA kits for CTGF are currently unavailable. Eleven of the 21 tumor samples assayed by ELISA contain TGF*β*1 protein (Figure 5), and seven of the 11 (64%) tumors are positioned downstream of tumor number 23. However, eight of the 21 assayed sarcomas are positioned upstream of tumor number 23, and four of the eight also contained TGF*β*1 protein. We found (1) the presence of TGF*β*1 protein within a tumor sample lacked any discernable association with that tumor TGF*β*1 transcript concentration and (2) TGF*β*1-bearing tumors did not cluster along the EGF-derived tumor continuum. These results suggest that TGF*β*1 translation or protein stability in sarcomas is probably not influenced directly by EGF, even though TGF*β*1 contribution to the Fas pathway may be influenced directly or indirectly by EGF. 

**Figure 6:**
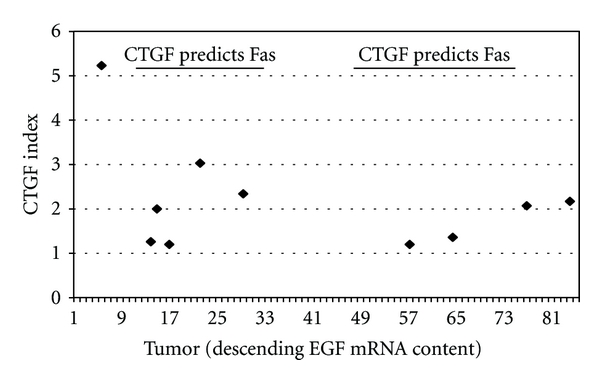
Sarcoma samples containing CTGF protein based on a dot blot analysis. The lines labeled “CTGF predicts Fas” overlay the tumors within which CTGF significantly predicted Fas mRNA content. Refer to Results and Discussion for derivation of CTGF Index.

A dot blot analysis documented the presence of CTGF protein in 10 of the 21 sarcoma samples (Figure 6). We blotted undiluted total soluble proteins from each of the 21 sarcoma samples and probed the dots with an anti-CTGF antibody. To normalize the data relative to each sample total soluble protein, each dot was scored numerically based on a visual estimate of dot intensity. The resulting numeric scores were divided by the known concentration of total protein within each sample. This procedure generated a “CTGF index.” The ordinate in Figure 6 represents the CTGF index, while tumor (ranked by descending EGF mRNA content) is represented on the abscissa. We also included within Figure 6 the range of tumors whose CTGF mRNA content predicted Fas transcript content (shown as a dark line in Figure 6). CTGF protein predominated in the tumors whose CTGF mRNA content served as the best predictor of Fas mRNA content. Thus, the dot blot results lend additional credence to our premise that CTGF associates with Fas.

### 3.7. Mechanism of Action

Our preliminary data and recent publications support our hypothesis that viable sarcoma cells regulate the Fas death pathway by activating EGF, which in turn influences the activities of TGF*β*1 and CTGF. Precedence for this hypothesis was provided by Reinehr et al. in 2008 [72]. We suggest inhibition of the Fas death pathway by CTGF in mesenchymal cells may occur directly (i.e., by binding to and thereby blocking the activities of key proteins), or indirectly, through CTGF association with EGF- or Fas-related pathways. CTGF, like other members of the CCN family, is composed of four conserved sequence motifs that are considered to be highly interactive with other biomolecules [76]. Although a number of proteins reportedly bind to CTGF [77, 78], with the exception of TGF*β*1, none of the binding partners reported to date are recognized components of the Fas death pathway. Therefore, it is unlikely that the inhibitory activities of CTGF result directly from physical contact with Fas-related proteins, or even through competition with relevant proteins. 

Although we can only speculate at this time, a likely scenario involves the association of CTGF with Fas- or EGFR-associated pathways. A candidate EGFR pathway involves c-Jun NH2-terminal kinase (JNK). Chen et al. [79] reported recently that cancer cells in general depend on constitutive activity of Fas, stimulated by FasL, for optimal growth and that the tumorigenic activity of Fas is mediated by a JNK. JNK is known to induce CTGF expression, typically via TGF*β*, in a variety of cell systems [80, 81]. CTGF subsequently activates MAPK/ERK [82–84], and activated MAPK/ERK blocks Fas-induced apoptosis [85]. Unfortunately the mechanism(s) involved in the MAPK inhibition of Fas-induced apoptosis are unknown [86]. Therefore, based on the literature, we propose that proteins involved with the ECM [87] may function in concert with CTGF and MAPK/ERK to block Fas-induced apoptosis; CTGF binds to integrin *α*_v_ *β*_3_ [88] and, when coupled with activated MAPK/ERK, ultimately promotes cell survival [89]. 

The study has substantial limitations. Tissue culture and *in vitro* data is anything but conclusive in terms of its relevance to the clinical situation, yet we hope this work will spur other efforts to look into this highly relevant pathogenic pathway with more robust modeling. This cohort represents a heterogenous group of rare tumors which generates a great detail of data noise and variability which can corrupt our analysis. However, the common theme across the cohort is that they are mesenchymal-derived neoplasms. In terms of microenvironment effect, gene expression profiles are certainly highly, and perhaps too, sensitive. As Heisenberg postulated in 1927, it is almost impossible to measure anything without affecting it.

## 4. Conclusion

To the best of our knowledge, this is the first data set in sarcomas detailing a potential growth factor/mitiogenic effect on FasL-mediated apoptosis. Although historically EGF has not been associated with cells of mesenchymal origin, we have documented in previous studies and in the current analysis an apparent direct relationship between EGF and the transcription of selected Fas-associated genes. We suggest the activities of other growth factors, such as TGF*β*1 and CTGF, may be regulated (or at least initiated) by EGF in mesenchymal cells. Our tumor and *in vitro* data strongly support a role for EGF modulation of Fas-induced cell death, probably through activation of CTGF. Mechanistically, CTGF may be induced by EGF in response to FasL stimulation. CTGF in turn activates a relevant pathway(s) (i.e., MAPK/ERK), and the activated pathway(s) blocks Fas-induced apoptosis. Further proteomic and *in vivo* studies will be necessary to substantiate this theory. However the basic tenet, derived from a consensus within the literature, is that CTGF is principally a prosurvival protein. This scenario therefore represents a novel role for EGF and CTGF as regulators of Fas-induced cell death in mesenchymal neoplasms. 

## Figures and Tables

**Table 1:** Coexpression matrix for 12 Fas-related genes assayed by RT-PCR in 82 primary sarcomas. Correlation coefficients in bold are significant at *P* ≤ 8.0*E* − 05 using the Bonferroni adjustment for multiple comparisons.

	EGF	FADD	DAXX	TGF*β*1	CTGF	Fas	SMAD3	SMAD7	CASP8	CASP10	CFLAR	FasL
EGF	1											
FADD	−0.03	1										
DAXX	0.06	**0.57**	1									
TGF*β*1	−0.08	0.27	0.37	1								
CTGF	0.11	0.25	0.37	**0.62**	1							
Fas	0.17	0.16	**0.44**	**0.57**	**0.66**	1						
SMAD3	0.12	0.20	**0.55**	**0.46**	0.41	**0.52**	1					
SMAD7	−0.02	0.12	0.33	**0.48**	**0.50**	**0.53**	**0.51**	1				
CASP8	−0.14	0.31	**0.54**	**0.45**	0.30	**0.49**	**0.61**	**0.54**	1			
CASP10	0.03	0.35	**0.43**	**0.58**	**0.48**	**0.52**	**0.50**	**0.49**	**0.69**	1		
CFLAR	−0.06	0.31	**0.49**	**0.43**	0.36	**0.43**	**0.52**	**0.53**	**0.80**	**0.71**	1	
FasL	0.04	0.03	−0.03	0.09	0.35	0.23	−0.07	−0.02	0.01	0.17	−0.07	1

**Table 2:** Coexpression matrices for nine Fas-related genes assayed by RT-PCR in 23 complete medium control (top panel) and 18 serum-free (SF) control (bottom panel) SW1353 chondrosarcoma cell cultures. Correlation coefficients in bold are significant at *P* ≤ 1.1*E* − 03 (Bonferroni's adjustment).

	EGF	TGF*β*1	CTGF	Fas	SMAD3	SMAD7	CASP8	CASP10	CFLAR
EGF	1	**0.85**	**0.73**	**0.63**	**0.67**	−0.17	0.25	0.19	0.16
TGF*β*1	0.26	1	**0.91**	**0.84**	**0.90**	−0.16	0.31	0.18	0.18
CTGF	0.29	**0.73**	1	**0.97**	**0.95**	−0.30	0.10	−0.05	−0.03
Fas	0.36	**0.92**	**0.77**	1	**0.94**	−0.41	−0.07	−0.22	−0.19
SMAD3	0.17	**0.84**	0.55	**0.88**	1	−0.20	−0.21	−0.04	−0.01
SMAD7	0.60	0.18	0.35	0.13	−0.07	1	0.60	**0.64**	**0.66**
CASP8	0.26	0.03	−0.15	−0.02	0.00	0.51	1	**0.95**	**0.94**
CASP10	0.42	0.22	−0.01	0.18	0.10	0.52	**0.92**	1	**0.98**
CFLAR	0.21	0.35	0.14	0.23	0.14	0.58	**0.86**	**0.90**	1

**Table 3:** Coexpression matrix for nine Fas-related genes assayed by RT-PCR in 34 SW1353 chondrosarcoma cell cultures treated topically with rhEGF, alone or following EGF/TGF*β* receptor inhibition. Correlation coefficients in bold are significant at *P* ≤ 1.1*E* − 03 (Bonferroni's adjustment).

	EGF	TGF*β*1	CTGF	Fas	SMAD3	SMAD7	CASP8	CASP10	CFLAR
EGF	1								
TGF*β*1	0.18	1							
CTGF	0.22	0.50	1						
Fas	0.28	**0.87**	**0.52**	1					
SMAD3	0.42	0.47	0.36	**0.62**	1				
SMAD7	**0.66**	0.23	0.17	0.27	−0.07	1			
CASP8	**0.76**	0.26	0.18	0.38	**0.54**	0.43	1		
CASP10	**0.74**	0.30	0.13	0.38	**0.57**	0.34	**0.96**	1	
CFLAR	**0.57**	0.45	0.12	0.50	0.33	**0.56**	**0.80**	**0.78**	1

**Table 4:** SW1353 chondrosarcoma cell cultures respond to rhEGF (1 ng/mL for 1 h) stimulation under serum-free culture conditions. Significant correlations are in bold.

Gene pair	Control (*n* = 10)	rhEGF (*n* = 10)
TGF*β*1:Fas	0.63	**0.92**
TGF*β*1:SMAD3	0.67	**0.96**
CTGF:Fas	0.43	**0.91**
SMAD3:Fas	0.70	**0.85**
EGF:SMAD7	0.67	**0.96**
TGF*β*1:CTGF	−0.17	0.80

**Table 5:** Predictive value of EGF, TGF*β*1, CTGF, and FasL for Fas, CASP8, and CASP10 mRNA concentrations in sarcomas and in serum-free (SF) and complete medium (CM) control and rhEGF-treated SW1353 chondrosarcoma cell cultures. Refer to Section 3.3 for separation of tumors into “top 41” and “bottom 41” tumor subcohorts. Significant probability values are in bold.

Samples	Response gene	Predictor genes			
		EGF	TGF*β*1	FasL	CTGF
82 tumors	Fas	0.11	0.09	**5.0*E *− 03**	**1.8*E *− 04**
Top 41 tumors	Fas	**2.0*E*−03**	0.48	**9.0*E *− 04**	0.01
Bottom 41 tumors	Fas	0.75	0.26	0.15	**4.0*E *− 04**
82 tumors	CASP8	0.21	0.03	0.06	0.94
Top 41 tumors	CASP8	0.34	**3.5*E *− 03**	**9.3*E *− 03**	0.96
Bottom 41 tumors	CASP8	0.13	0.89	0.35	0.50
82 tumors	CASP10	0.70	0.01	**1.4*E *− 04**	0.38
Top 41 tumors	CASP10	0.36	**2.9*E *− 03**	0.04	0.92
Bottom 41 tumors	CASP10	0.62	0.88	**5.9*E *− 03**	0.11
SW1353 SF control	Fas	0.31	**7.4*E *− 05**	n/a	0.22
SW1353 CM control	Fas	0.41	0.57	n/a	**9.5*E *− 06**
SW1353 rhEGF	Fas	0.24	**6.8*E *− 09**	n/a	0.33
SW1353 SF control	CASP8	0.23	0.47	n/a	0.25
SW1353 SF control	CASP10	0.09	0.20	n/a	0.19
SW1353 CM control	CASP8	0.50	**2.9*E *− 03**	n/a	**2.8*E *− 03**
SW1353 CM control	CASP10	0.80	**1.1*E *− 03**	n/a	**3.6*E *− 04**
SW1353 rhEGF	CASP8	**6.3*E *− 07**	0.28	n/a	0.70
SW1353 rhEGF	CASP10	**1.0*E *− 06**	0.09	n/a	0.29
